# Arsenic and Latent Disease Risk: What’s the Mechanism of Action?

**DOI:** 10.1289/ehp.124-A36

**Published:** 2016-02-01

**Authors:** Carol Potera

**Affiliations:** Carol Potera, based in Montana, also writes for *Microbe*, *Genetic Engineering News*, and the *American Journal of Nursing*.

There is evidence that people exposed early in life to arsenic-tainted drinking water may be at risk for a variety of diseases in adulthood. For instance, studies of Chileans exposed to contaminated water prenatally or in childhood showed increased risk of death from kidney, lung, and liver cancers by their 30s and 40s.^1^ However, the mechanisms by which arsenic may act are obscured by the complexity of these and other diseases and exposures involved. A commentary in this issue of *EHP* discusses potential molecular mechanisms by which arsenic may induce latent diseases that appear long after exposure.^2^

One hypothesis discussed is that epigenetic changes, such as DNA methylation, alter gene expression and increase disease susceptibility. In one study, newborn mice exposed to arsenic levels known to cause liver cancer showed altered DNA methylation and expression of genes related to the disease.^3^ These changes may be sex-dependent, with another study reporting changes in methylation in Bangladeshi boys exposed prenatally to arsenic, but not in girls.^4^

**Figure:**
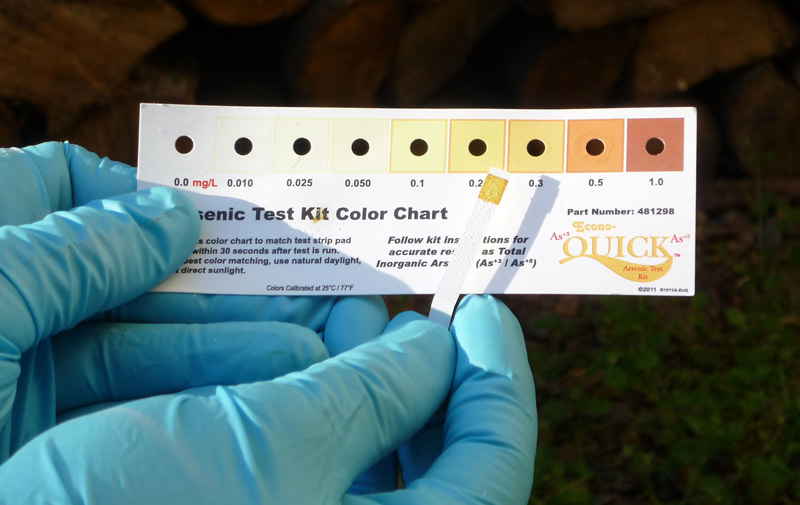
Although we still don’t fully understand exactly how arsenic causes cancer and other adverse health effects, researchers advise that people can and should take practical steps to avoid exposure, such as testing their well water for contamination. Courtesy of Robert Marvinney/Maine Geological Survey

It’s unknown whether arsenic-induced epigenetic changes are transgenerational—in other words, whether nonexposed children and grandchildren of arsenic-exposed individuals also may be at increased risk of cancer. “It would be difficult and expensive to monitor this in people,” says lead commentary author Rebecca Fry, an associate professor at the University of North Carolina at Chapel Hill. Mouse studies could track effects more quickly through multiple generations, but that, too, would be costly, Fry says.

Cancer stem cells (CSCs) represent another new and understudied area of great interest. CSCs are hypothesized to arise from normal stem cells and to be the force that enables tumors to grow and spread.^5^ CSCs remain dormant in the target organ until unknown triggers incite them to transform into cancer cells.^2^ Aberrant gene expression may be one such trigger, as suggested by mouse studies in which prenatal arsenic exposure was associated with increased expression of several cancer-related genes, along with highly aggressive squamous cell skin cancers.^6^

A third proposed mechanism is that arsenic targets the immune system. The authors cite multiple studies that have identified altered immune factors associated with either immunosuppression or inflammation in the cord blood of newborns exposed *in utero* to arsenic. If immune disruption were to persist after birth—a possibility that remains untested—the result could be increased susceptibility to infection and/or a variety of chronic diseases.^2^

“This collective effort provides a timely update of the possible mechanisms by which early-life arsenic exposure may increase risk of diseases such as cancer,” says Margaret Karagas, chair of the Department of Epidemiology at the Geisel School of Medicine at Dartmouth. Karagas, who was not involved with the commentary, suggests future investigations should focus on how arsenic exposure during precise windows in gestation and early life impact disease risk later. She also points to the growing body of literature on dietary sources of arsenic, which she says will need to be considered in strategies to reduce arsenic-induced disease.

Given that more than 200 million people worldwide drink water containing arsenic that exceeds the World Health Organization’s guideline of 10 µg/L,^7^ Fry proposes making better use of what’s already known while researchers continue to study arsenic’s molecular mechanisms. One practical step forward would be for pregnant women and those considering pregnancy to test their drinking water for arsenic and switch to an alternative source if contamination is detected. “It’s a gap in public health strategy,” Fry says, “that arsenic findings have not been translated to the clinic.”
